# Patient-derived glioblastoma stem cells are killed by CD133-specific CAR T cells but induce the T cell aging marker CD57

**DOI:** 10.18632/oncotarget.2767

**Published:** 2014-11-15

**Authors:** Xuekai Zhu, Shruthi Prasad, Simone Gaedicke, Michael Hettich, Elke Firat, Gabriele Niedermann

**Affiliations:** ^1^ Department of Radiation Oncology, University Hospital Freiburg, Freiburg, Germany; ^2^ Faculty of Biology, University of Freiburg, Freiburg, Germany; ^3^ German Cancer Consortium (DKTK), Freiburg, and German Cancer Research Center (DKFZ), Heidelberg, Germany

**Keywords:** chimeric antigen receptor, cancer stem cell, glioblastoma, immunotherapy, CD57

## Abstract

The AC133 epitope of CD133 is a cancer stem cell (CSC) marker for many tumor entities, including the highly malignant glioblastoma multiforme (GBM). We have developed an AC133-specific chimeric antigen receptor (CAR) and show that AC133-CAR T cells kill AC133+ GBM stem cells (GBM-SCs) both *in vitro* and in an orthotopic tumor model *in vivo*. Direct contact with patient-derived GBM-SCs caused rapid upregulation of CD57 on the CAR T cells, a molecule known to mark terminally or near-terminally differentiated T cells. However, other changes associated with terminal T cell differentiation could not be readily detected. CD57 is also expressed on tumor cells of neural crest origin and has been preferentially found on highly aggressive, undifferentiated, multipotent CSC-like cells. We found that CD57 was upregulated on activated T cells only upon contact with CD57+ patient-derived GBM-SCs, but not with conventional CD57-negative glioma lines. However, CD57 was not downregulated on the GBM-SCs upon their differentiation, indicating that this molecule is not a *bona fide* CSC marker for GBM. Differentiated GBM cells still induced CD57 on CAR T cells and other activated T cells. Therefore, CD57 can apparently be upregulated on activated human T cells by mere contact with CD57+ target cells.

## INTRODUCTION

Many malignant tumors appear to contain a subpopulation of stem-like cells commonly termed cancer stem cells (CSCs). In contrast to more differentiated tumor cells, CSCs are thought to be crucial for the initiation and long-term propagation of tumors, owing to their high self-renewal capacity. This property and their high resistance to conventional chemo- and radiotherapy are thought to significantly contribute to the formation of tumor recurrences and metastases. In addition, there is evidence that CSCs drive tumor invasion and are immunosuppressive [1, 2]. CD133-positive CSCs have been found in glioblastoma multiforme (GBM) and other brain tumors, and their frequency appears to increase at recurrence [1, 3]. GBM is a fast growing and highly invasive tumor entity. It is the most aggressive primary brain tumor in adults, with a median survival time of 15–17 months [4], and remaining CSCs are likely to be an important factor impeding complete cures upon standard therapy with surgery and radiochemotherapy [5]. Factors contributing to the resistance of CSCs to conventional genotoxic therapies include overexpression of drug efflux proteins, DNA repair and anti-apoptotic proteins, and elevated levels of antioxidants. In addition, in some but not all tumor entities, CSCs are believed to be largely quiescent, and quiescence is an important resistance factor to genotoxic therapies [1, 2, 6, 7]. Cellular immunotherapies may be able to eliminate therapeutically resistant CSCs because immune-mediated killing is not influenced by most of the aforementioned factors.

Adoptive transfer of T cells genetically modified to express chimeric antigen receptors (CARs) has recently shown unexpectedly high therapeutic activity in late-stage hematological malignancies [8, 9]. Therapeutic activity has also been observed in some patients with solid tumors [8, 10]. CARs are artificial T cell receptors (TCRs), in which an extracellular binding domain of known antibodies or ligands is fused with the intracellular signaling domain of the CD3 ζ chain of the physiological TCR, without or in tandem with signaling domains of co-stimulatory receptors such as CD28, CD137 (4-1BB), CD134 (OX40), or CD278 (ICOS) to supply additional signals for T cell activation, proliferation, and survival. Whereas the first-generation CARs did not contain co-stimulatory signaling domains, the second- and third-generation CARs contain one or two of these domains, respectively [8, 9, 11]. So far, CAR transgenes have been integrated into T cells by using recombinant retroviral (gamma-retroviral or lentiviral) vectors or nonviral (*sleeping beauty* or *piggyBac* transposon/transposase) systems. The retroviral system has been widely used for producing clinical-grade CAR T cells. However, the transposon system has several potential advantages: a shorter time and lower cost for the manufacture of CAR T cells because the virus production step can be avoided, and a high capacity for expressing foreign genes in CAR T cells because transposons can carry larger transgenes than retroviruses and because more than one transposon can be efficiently delivered into T cells at the same time [9, 12].

Strong effects have already been reported for CAR T cells specific for the putative CSC antigens CD20 or CD24 against melanoma [13] and pancreatic adenocarcinoma [14] xenografts, respectively, in which just a subpopulation of the tumor cells expressed the targeted antigens. The results fit well to the CSC hypothesis, according to which only CSCs but not the more differentiated tumor cells are responsible for long-term tumor propagation. In GBM models, CARs targeting the human epidermal growth factor receptor 2 (HER2) [15], the epidermal growth factor receptor variant III (EGFRvIII) [16], or the IL-13 receptor α2 (IL13Rα2) [17] have been shown to be effective against GBM stem cell (GBM-SC) lines in preclinical *in vitro* or *in vivo* models. However, none of these markers is expressed in all GBMs and, in addition, the SC populations in most GBMs (and in other tumor entities) are probably heterogeneous in terms of surface marker expression. It is thus important to develop more CARs with specificity for CSCs including GBM-SCs.

In this work, we engineered human CD8+ T cells to target CD133-positive CSCs. The transmembrane glycoprotein CD133/prominin is *per se* not stem cell specific. However, AC133, an N-glycosylation-dependent epitope of CD133, is almost exclusively stem cell specific. The epitope has been described as a CSC marker for a large variety of brain and extracranial tumors and is regarded as a prototypic GBM-SC marker [3, 18]. We generated AC133-specific CAR T cells by stable transfection with a third-generation CAR containing an AC133-specific single-chain antibody (scFv) using the *PiggyBac* transposon/transposase system. AC133-CAR T cells were able to kill patient-derived GBM-SCs *in vitro* with high specificity and they prolonged the survival of immunodeficient mice with established orthotopic brain tumors initiated from patient-derived GBM-SCs. In addition, we report the new finding that, upon contact with patient-derived GBM cells, CAR T cells or nontransfected activated human CD8+ T cells display high expression of CD57, a molecule that, when expressed on T cells, is best known to mark terminally differentiated (i.e., senescent) T cells [19, 20]. However, other typical changes of end-stage T cell differentiation were not detected, even not after further short-term stimulation, and we obtained evidence that mere contact between patient-derived GBM-SCs and T cells is sufficient to upregulate CD57 on activated T cells. Interestingly, all the tested patient-derived GBM-SC lines themselves turned out to be positive for CD57, which has also been described to be enriched in undifferentiated neuroblastoma [21] and Ewing sarcoma cells with CSC features [22]. However, we found that differentiated tumor cells originating from patient-derived GBM-SCs still express CD57, so that CD57 appears not to be a *bona fide* CSC marker for GBM.

## RESULTS

### Development of third-generation CAR T cells targeting the CSC marker AC133

**Figure 1:**
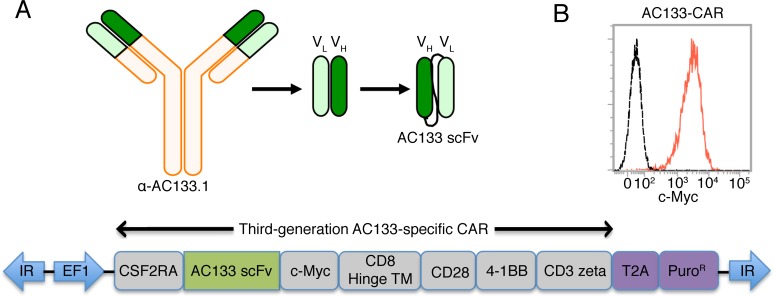
Scheme of the AC133-specific CAR and flow cytometric characterization of AC133-CAR expression (A) The AC133 scFv was derived from the anti-human AC133.1 antibody. The cDNA of the third-generation CAR is composed of sequences coding for the signal peptide from colony-stimulating factor-2 receptor alpha (CSF2RA), the AC133 scFv, a c-Myc tag, the hinge and transmembrane regions from human CD8, and the signaling domains from human CD28, 4-1BB, and CD3 zeta. The coding sequence for the puromycin resistance marker (Puro^R^), preceded by a “self-cleaving” 2A peptide (T2A), was placed after the CAR coding sequence. The inverted terminal repeats (IR) and the EF1 alpha promoter (EF1) originate from the *piggyBac*-transposon vector. (B) AC133-CAR expression on T cells was determined according to flow cytometric detection of the c-Myc tag. AC133-CAR T cells were stained with mouse anti-c-Myc, followed by a PE-labeled rabbit anti-mouse secondary antibody. Unstained T cells were used as control (dashed). Data shown are representative of several different experiments.

We generated AC133-CAR-expressing T cells by nucleofection of transposon vectors [12] containing a third-generation CAR. As shown in Figure 1A, the AC133 scFv was derived from the anti-AC133.1 mAb [23]. The third-generation CAR cDNA was gene-synthesized and inserted into a commercial *piggyBac* transposon vector. Then, the transposon and transposase plasmids were nucleofected together into peripheral blood mononuclear cells (PBMCs). Selection for CAR expression with puromycin and expansion in T cell medium with the anti-CD3 antibody OKT3 and 40-Gy γ-irradiated allogeneic PBMCs as feeder cells were conducted. On day 24, almost all the T cells expressed AC133-specific CARs, as shown in Figure 1B, detected by anti-c-Myc antibody.

### AC133-specific CAR T cells recognize and kill patient-derived glioma cells

**Figure 2:**
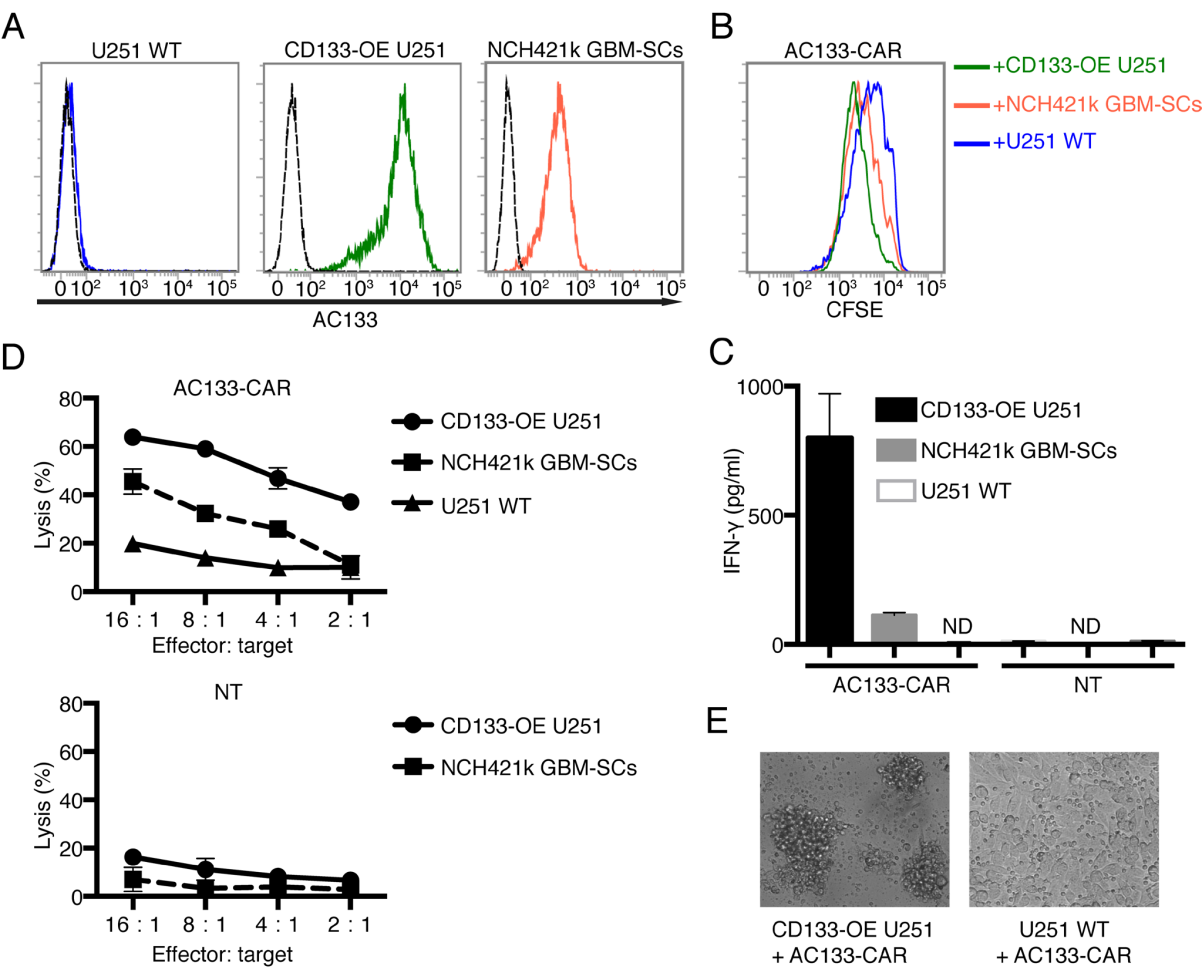
AC133-CAR T cells specifically recognize and kill AC133-positive glioma cells *in vitro* (A) Flow cytometric analysis of AC133 expression on the target cells used in this study (AC133-negative U251 WT cells, AC133+ CD133-OE U251 glioma cells, and AC133+ patient-derived NCH421k GBM-SCs). (B) CAR T cell proliferation induced upon contact with different target cells. AC133-CAR T cells were labeled with CFSE and then co-cultured with the indicated types of irradiated target cells for 4 days. The extent of T cell proliferation is reflected by the loss of incorporated CFSE and correlates with AC133 expression on the target cells. (C) IFN-γ secretion by CAR T cells upon contact with different target cells. AC133-CAR T cells or nontransfected T cells (NT) were co-cultured with different target cells for 24 h. Thereafter, culture supernatants were collected and analyzed for the concentration of IFN-γ by ELISA. AC133-CAR T cells produced IFN-γ upon stimulation with AC133+ tumor cells. No appreciable cytokine release was detected in cultures with nontransfected control T cells. ND, below the limit of detection. (D) Flow cytometric PKH-26 assay for the determination of target cell lysis. AC133-positive CD133-OE U251 glioma cells and NCH421k GBM-SCs were lysed by AC133-CAR T cells. U251 WT target cells and nontransfected T cells were used as negative controls. (E) Microscopical images after 24-h co-culture of CAR T cells with AC133+ and AC133– target cells, respectively. Whereas AC133– U251 WT glioma cells still adhered to the culture plates, the AC133+ CD133-OE U251 glioma cells had detached and formed clumps with the attacking AC133-CAR T cells. Data shown are representative of at least three independent experiments. In (C) and (D), data are shown as mean ± SD from triplicate experiments.

Next, we investigated whether AC133-CAR-expressing T cells could recognize AC133-positive glioma cells *in vitro*. We used three different glioma lines: AC133/CD133-negative U251 wild-type (WT) cells, used as a control; CD133-overexpressing (OE) U251 cells transduced with a CD133-encoding lentivirus [24]; and the patient-derived GBM-SC line NCH421k [25] with an approximately 10–15-fold lower AC133 expression compared to the CD133-OE U251 glioma line (Figure 2A). Incubation with AC133+ tumor cells caused activation of the AC133-specific CAR T cells, leading to enhanced T cell division, as indicated by a cell division assay using the cell tracker dye carboxyfluorescein diacetate succinimidyl ester (CFSE). The extent of CFSE decrease correlated with the level of AC133 expression on the tumor cells (Figure 2B). In addition, IFN-γ secretion by the CAR T cells also correlated with the AC133 expression level on the tumor cells (Figure 2C). These assays indicated that the CAR T cells specifically recognized the AC133-positive glioma cells, including the patient-derived GBM-SCs which exhibit a rather low AC133 expression. This was also reflected in cytotoxicity assays. AC133-specific CAR T cells killed AC133-positive glioma cells, and the extent of killing correlated with both the AC133 expression level on the tumor cells and the CAR T cell/tumor cell ratio (Figure 2D, upper panel). Nontransfected T cells did not kill glioma cells (Figure 2D, lower panel). The specific killing was also evident by microscopic examination (Figure 2E). Co-incubation of AC133-specific CAR T cells with AC133-negative WT U251 cells caused neither microscopic signs of T cell activation nor of tumor cell killing (Figure 2E, right). In contrast, co-incubation with either AC133+ CD133-OE U251 cells (Figure 2E, left) or NCH421 GBM-SCs (not shown) caused detachment of the target cells and rosette formation, indicating clumping of antigen-expressing tumor cells with AC133-specific CAR T cells and proliferation of the latter.

### AC133-specific CAR T cells inhibit the growth of orthotopic xenografts initiated from GBM-SCs

**Figure 3:**
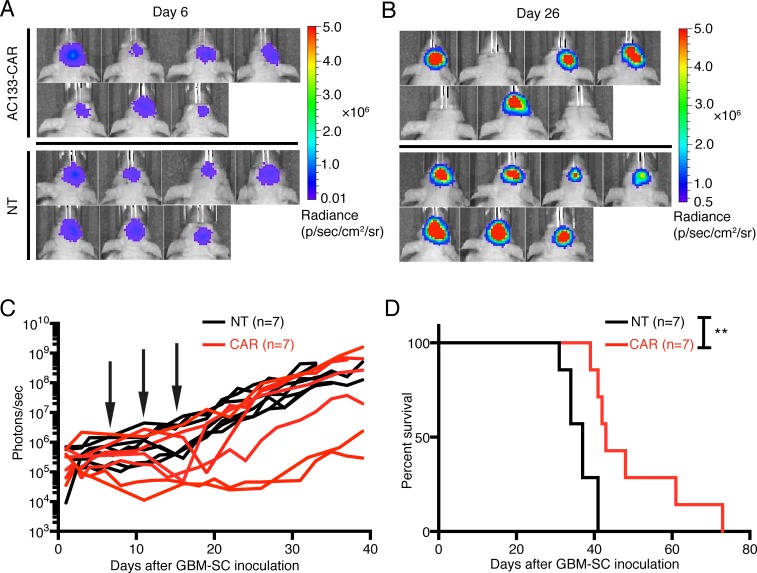
AC133-CAR T cells show therapeutic efficacy against established, orthotopic GBM-SC-derived glioma xenografts in mice Firefly luciferase-expressing AC133+ NCH421k GBM-SCs (1 × 10^5^) were injected into the forebrains of nude mice. On days 7, 11, and 15 of tumor growth, 2 × 10^6^ AC133-specific CAR T cells or nontransfected T cells were injected via the same coordinates. (A, B) Bioluminescence images taken before (day 6, A) and after (day 26, B) the treatments with either AC133-specific CAR T cells or nontransfected control T cells. (C) Summary of all BLI measurements. Arrows, time of T cell injection; n, number of animals tested in each group. (D) Kaplan-Meier analysis revealed a statistically significant longer survival of the AC133-CAR T cell-treated mice compared to mice treated with nontransfected control T cells (*p* <0.003).

We used an orthotopic xenograft tumor model in athymic nude mice for the treatment of established gliomas initiated from patient-derived GBM-SCs [24]. As tumor cells, we used AC133+ NCH421k GBM-SCs transduced with the latest generation of firefly luciferase. The use of luciferase-expressing tumor cells allowed the non-invasive monitoring of tumor growth by bioluminescence imaging (BLI; Figure 3A–C). The treatment with CAR T cells was started at day 7 after tumor cell inoculation. The CAR T cells were injected intracerebrally (i.c.) into the tumor region, and the treatment was repeated on days 11 and 15 after tumor cell inoculation. As shown in Figure 3A, B (bottom), and C (black lines), after treatment with nontransfected control T cells, the tumor BLI signal in all mice continued to increase. In contrast, administration of AC133-CAR T cells caused a strong long-lasting delay of the BLI signal increase in 3 and a transient decrease of the BLI signal in 2 of 7 mice [Figure 3A, B (top), and C (red lines)]. This treatment effect was reflected in a significantly increased overall survival of the mice treated with the AC133-specific CAR T cells (Figure 3D).

### NCH421k GBM-SCs induce the upregulation of CD57 on CAR T cells

In adoptive transfers of antitumoral T cells, highest activity has been ascribed to minimally differentiated (non-senescent) and non-exhausted T cells [26, 27]. However, chronic T cell stimulation as it occurs after transfer of CAR T cells into hosts with high tumor load, or as may also occur during *in vitro* CAR T cell expansion, can lead to terminal differentiation (replicative senescence with loss of proliferative capacity, but high cytotoxic activity) or exhaustion (functional impairment with subsequent physical deletion) of T cells, or both [28-30]. In addition, CSCs have been reported to suppress T cells [31, 32], which can be reflected by an inhibition of T cell proliferation and activation as well as an increase in apoptotic T cells. We therefore wanted to know whether our CAR T cell manufacturing protocol or the encounter with AC133+ GBM-SCs induce an impairment of AC133-specific CAR T cells. For this purpose, we assessed the expression levels of the T cell exhaustion marker programmed death 1 (PD-1) and the terminal differentiation/senescence marker CD57 [19, 20, 28].

**Figure 4:**
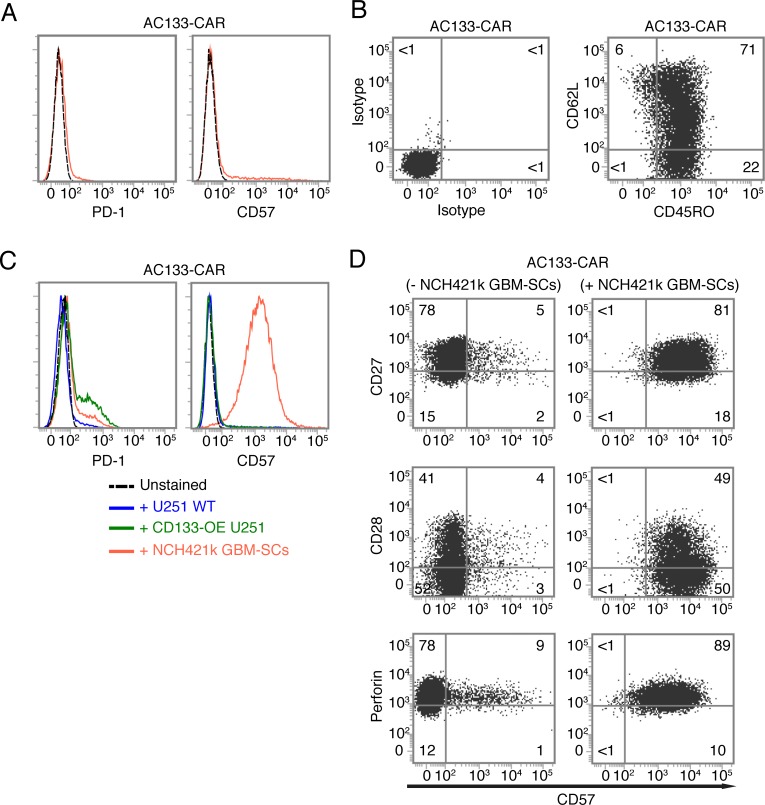
Expression of T cell exhaustion and senescence markers on AC133-CAR T cells (A) Near absence of the exhaustion marker PD-1 and the senescence marker CD57 on AC133-CAR T cells after two rounds of expansion with anti-CD3, IL-2, and feeder cells. (B) T cell differentiation marker expression after two rounds of expansion: CAR T cells were stained with anti-CD62L-APC and anti-CD45RO-FITC (right) or matched isotype control antibodies (left). Most of the cells had a central memory phenotype (CD45RO+ CD62L+). Data shown are representative of at least three individual experiments. (C) A 24-h co-culture with NCH421k GBM-SCs, but not with CD133-OE or WT U251 cells, causes strong upregulation of CD57 on CAR T cells; PD-1 was slightly upregulated upon co-culture with NCH421k or CD133-OE U251 cells. (D) AC133-CAR T cells, cultured for 24 h either alone or with NCH421k GBM-SCs, were stained with anti-CD57 and analyzed for co-expression of either CD27 (top), CD28 (middle), or perforin (bottom) by flow cytometry. Neither CD27 nor CD28 were downregulated, and perforin expression was not changed either. Data presented are representative of two individual experiments.

As shown in Figure 4A, CAR T cells expanded according to our protocol were negative for both PD-1 and CD57, indicating that our manufacturing protocol induces neither T cell exhaustion nor terminal T cell differentiation. This is consistent with the finding that ~70% of the CAR T cells expressed CD62L and CD45RO, a surface phenotype typical of central memory T cells [29], corresponding to an intermediate state of T cell differentiation (Figure 4B). We then analyzed potential phenotypic changes of the CAR T cells upon contact with different glioma cell lines. As shown in Figure 4C (left), PD-1 was only slightly upregulated on a fraction of the AC133-specific CAR T cells upon contacting antigen-positive tumor cells, and the degree of upregulation correlated with the level of AC133 expression on the target cells. Therefore, PD-1 upregulation appeared to depend on specific antigen recognition and (re)stimulation of the T cells, and not on the differentiation state (stemness or differentiation) of the tumor cells. However, we observed a strong upregulation of CD57 on the CAR T cells upon co-culture with NCH421k GBM-SCs, but not upon co-culture with conventional U251 WT nor with CD133-OE U251 glioma cells (Figure 4C, right). Since CD57 is best known as a marker for late or terminally differentiated human T cells [19, 20], we then checked the expression of other markers related to terminal T cell differentiation. Besides an increase in CD57, terminal differentiation of human T cells is usually accompanied by loss of the positive co-stimulatory molecules CD27 and CD28 (correlating with critical telomere shortening and loss of telomerase activity) and an elevation of perforin expression, indicating the high cytotoxicity of terminally differentiated T cells [19, 28]. However, neither CD27 nor CD28 were downregulated on the CAR T cells upon co-culture with AC133+ NCH421k GBM-SCs, and the perforin levels did not change (Figure 4D). From these experiments, we concluded that CD57 upregulation on the CAR T cells upon encounter with the GBM-SCs did not reflect a terminally differentiated T cell state. CD57 upregulation on CD27+ T cells has recently been linked to a state of incomplete differentiation of tumor-infiltrating T cells (TILs), which, in contrast to terminally differentiated T cells, are still able to proliferate upon TCR stimulation but rapidly undergo terminal differentiation (indicated by the downregulation of CD27 and CD28 and the upregulation of perforin) upon further antigen stimulation [33]. After incubation with NCH421k GBM-SCs and subsequent upregulation of CD57, our AC133-specific CAR T cells still proliferated when further cultured on anti-CD3 antibody-coated plates or with AC133+ NCH421k GBM-SCs for several days and they downregulated neither CD27 nor CD28 (data not shown), suggesting that the strong CD57 upregulation on the CAR T cells upon incubation with GBM-SCs did not reflect terminal replicative senescence, nor was it identical to the state of incomplete T cell differentiation recently described by Wu *et al*. [33].

### Upregulation of CD57 on T cells is not dependent on CAR target recognition but requires cell-cell contact with CD57-positive GBM-SCs

**Figure 5:**
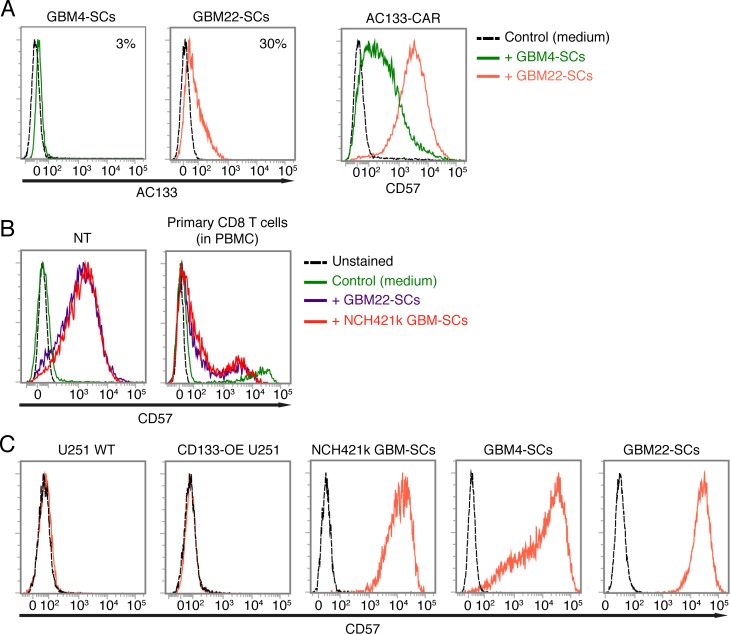
Upregulation of CD57 on AC133-CAR T cells or nontransfected activated T cells upon co-culture with CD57-positive GBM-SCs (A) The AC133-negative GBM-SC line GBM4 and the weakly AC133-positive GBM-SC line GBM22 (left) strongly induce CD57 on AC133-CAR T cells after a 24-h co-culture (right). (B) Patient-derived GBM-SCs also strongly induce the upregulation of CD57 on nontransfected anti-CD3-activated control T cells (left); in contrast, only a small subpopulation of non-CD3-activated, peripheral blood CD8 T cells expresses CD57, irrespective of co-incubation with GBM-SCs (right). (C) Flow cytometric analysis showing that all the GBM-SCs investigated in this study, but not the conventional U251 glioma cell line, express high levels of CD57. Data presented are representative of at least three independent experiments.

Since CD57 was upregulated on CAR T cells upon their encounter with AC133+ NCH421 GBM-SCs but not with conventional AC133+ CD133-OE-U251 or WT U251 glioma cells, we next wanted to know whether CD57 upregulation on the T cells depends on the differentiation state of the tumor target cells. For this purpose, we co-cultured the CAR T cells with two other GBM-SC lines, GBM4 and GBM22 [34], one of which is AC133 negative and the other one shows low AC133 expression, respectively (Figure 5A, left). As shown in Figure 5A (right), both GBM-SC lines could induce upregulation of CD57 on the CAR T cells. As these two GBM-SC lines express only little or no AC133, CD57 upregulation on the CAR T cells seems not to rely on specific antigen recognition. Therefore, we then investigated whether CD57 is also upregulated on nontransfected control T cells or primary CD8+ T cells within the PBMC population. Strong upregulation of CD57 was also found on nontransfected, activated control T cells (Figure 5B, left) but not on primary CD8+ T cells isolated from blood (Figure 5B, right), which contained a small proportion of CD57+ cells, independent of the incubation with the CSCs. Thus, CD57 upregulation on T cells may require prior T cell activation.

**Figure 6:**
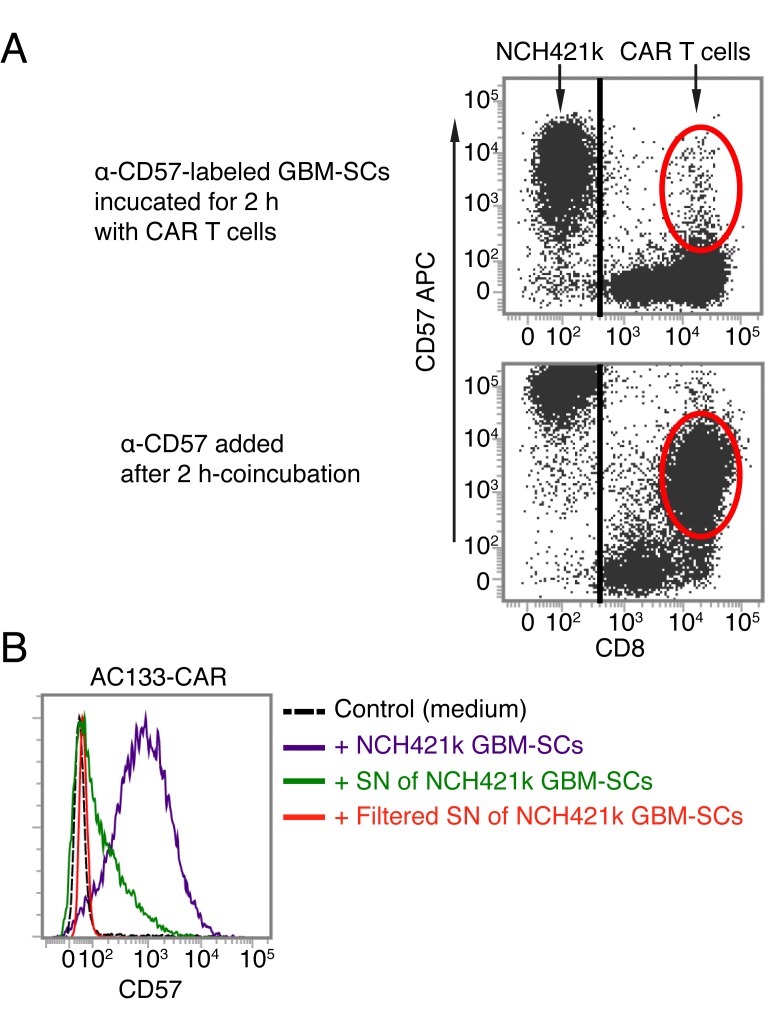
AC133-CAR T cells do not seem to directly acquire the CD57 epitope from GBM-SCs (A) CD57+ NCH421k GBM-SCs were stained with anti-CD57-APC, washed and then co-cultured with AC133-CAR T cells for 2 h. Thereafter, the APC signal was analyzed on the AC133-CAR T cells, which turned out to be virtually negative for APC (top), suggesting that the epitopes or proteins bearing the CD57 epitope are not directly transferred from the GBM-SCs to the activated T cells. In a separate sample (bottom), the upregulation of CD57 on the AC133-CAR T cells was confirmed by staining with anti-CD57-APC after co-culture with the GBM-SCs. (B) Direct cell-cell contact between patient-derived GBM-SCs and activated T cells seems to be required for the upregulation of CD57 on the T cells, because GBM-SC supernatant (SN) only slightly induced CD57 on the T cells, and filtered GBM-SC supernatant, not at all. Data are representative of two independent experiments.

CD57 is a terminally sulfated glycan carbohydrate epitope, which is also expressed on certain proteins of cells of neural crest origin including the brain and peripheral nervous tissues [20, 35]. We therefore wanted to find out whether the glioma lines used in this study express CD57. As shown in Figure 5C, only the patient-derived GBM-SC lines, which induced the upregulation of CD57 on T cells, turned out to be CD57 positive. In contrast, the conventional glioma cell line U251, which did not induce CD57 upregulation on T cells, turned out to be CD57 negative. We therefore investigated whether the CAR T cells acquire the CD57 epitope from the patient-derived GBM-SCs. To assess this, we prelabeled the patient-derived GBM-SCs with a fluorescence-labeled anti-CD57 mAb (anti-CD57-APC), washed them thoroughly and thereafter incubated them with unstained CAR T cells for 2 h. As shown in Figure 6A (top), we could not detect the fluorescence-labeled anti-CD57 mAb on the CAR T cells after this co-incubation. In contrast, addition of APC-labeled anti-CD57 at the end of the 2-h co-incubation brightly stained the CAR T cells, again indicating a strong upregulation of CD57 on the CAR T cells upon co-incubation with the GBM-SCs (Figure 6A, bottom). Thus, CAR T cells do not seem to acquire CD57 directly from the GBM-SCs. Nevertheless, direct contact with the CD57-positive target cells is apparently required for the upregulation of CD57 on T cells, because transferring filtered medium from NCH421k GBM-SC cultures did not induce an upregulation of CD57 on T cells (Figure 6B).

### CD57 expressed on GBM-SCs seems not to be related to stemness

**Figure 7:**
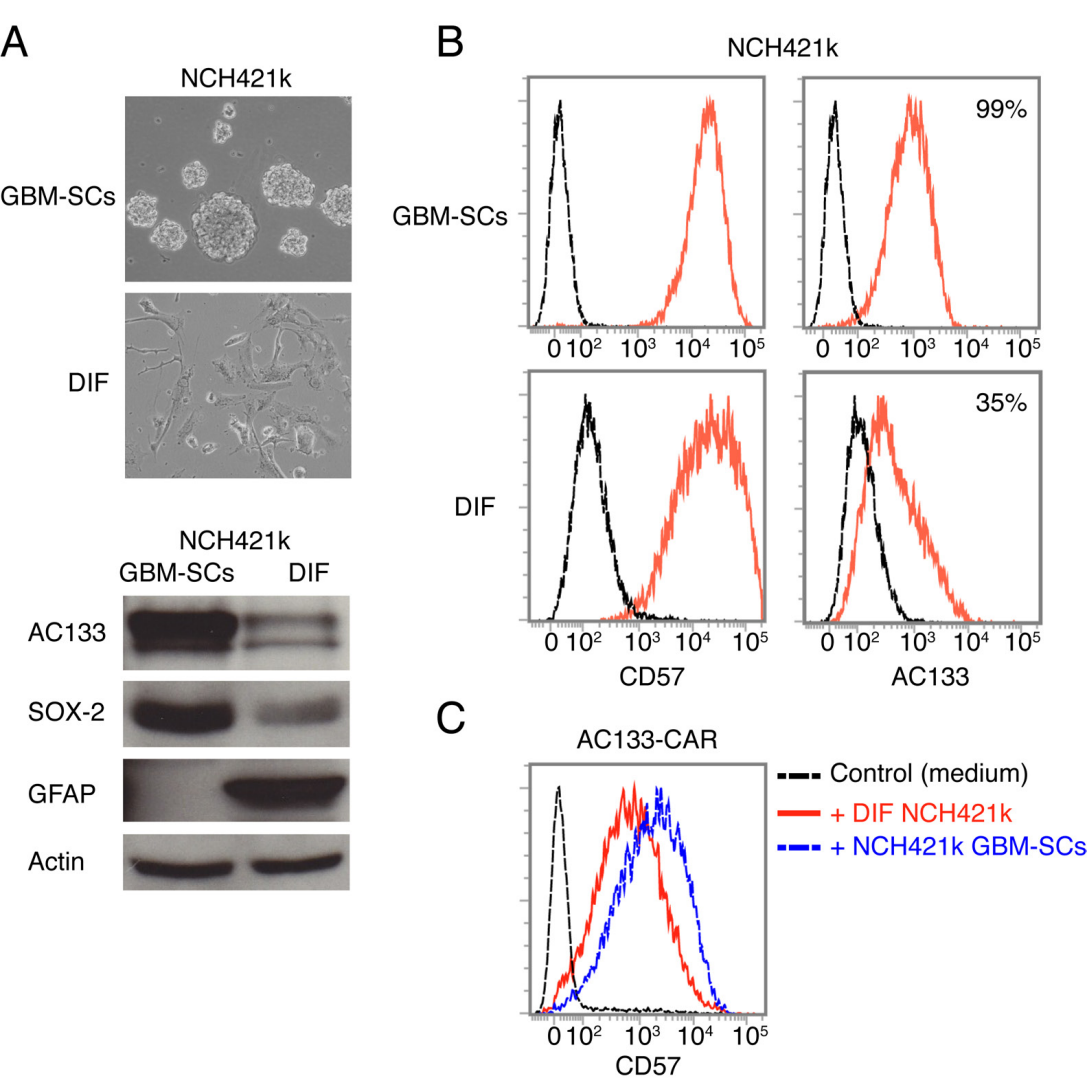
CD57 is not a bona fide CSC marker for GBM (A) Sphere-forming NCH421k GBM-SCs strongly change morphology and downregulate the well-known GBM-SC markers AC133/CD133 and Sox2, and upregulate the glial differentiation marker GFAP upon a 14-day culture in differentiation-inducing medium containing FCS and retinoic acid. (B) However, flow cytometry revealed that CD57 was not downregulated under these conditions. (C) The CD57-positive, differentiated NCH421k cells still induced upregulation of CD57 on AC133-CAR T cells. Data are representative of two individual experiments.

CD57 has been described to be preferentially expressed by undifferentiated neuroblastoma and Ewing sarcoma cells with stem-like features [21, 22]. Hence, we wanted to know whether CD57 is a CSC marker for GBM-SCs. For this purpose, we differentiated the GBM-SCs by withdrawal of the stem cell growth factors EGF and basic fibroblast growth factor (bFGF) and exposure to FCS-containing medium supplemented with retinoic acid [24, 25]. As shown in Figure 7A (top), after 2 weeks of exposure to differentiation-inducing factors, the small, floating, clustered GBM-SCs adhered and exhibited morphological features similar to those of differentiated brain cells. In addition, the cells had downregulated the well-known CSC markers AC133 and SOX2 and upregulated the glial differentiation marker GFAP (Figure 7A, bottom, and 7B, right). However, as shown in Figure 7B (left), CD57 was not downregulated on FCS/retinoic acid-differentiated glioma cells. The expression of CD57 on glioma cell lines derived under stem cell culture conditions seemed not to be related to stemness. We therefore conclude that CD57 does not serve as a CSC marker in GBM. CD57-positive cells differentiated from patient-derived GBM-SCs could still induce the upregulation of CD57 on CAR T cells (Figure 7C).

## DISCUSSION

We have developed a CAR with specificity for the prototypic CSC marker AC133 [3, 18] and provide evidence that AC133-CAR T cells eliminate patient-derived AC133+ GBM-SCs both *in vitro* and in an orthotopic glioma model *in vivo*. In addition, we wanted to address the issue of potential functional impairment of CAR T cells by CSCs. Surprisingly, we found that CD57, a marker that has been associated with terminal T cell differentiation [19, 20, 28] or incomplete differentiation (as a pre-stage of terminal T cell differentiation) [33], is strongly and rapidly upregulated on activated human (CAR) T cells upon direct contact with CD57+ patient-derived glioma cells but not with long-term passaged, conventional CD57-negative tumor cell lines. However, other phenotypic or functional signs of end-stage T cell differentiation were not detectable immediately or after further short-term T cell stimulation so that we conclude that activated T cells can upregulate CD57 by mere contact with CD57+ target cells. Moreover, CD57 upregulation on the activated T cells was unrelated to the stemness of the tumor cells, since differentiated patient-derived GBM cells, which kept CD57 expression, still induced CD57 expression on the activated T cells. The lack of CD57 downregulation on GBM-SCs upon their differentiation also suggests that CD57, which has been preferentially found on stem-like rather than differentiated Ewing sarcoma [22] and neuroblastoma cells [21], is not a *bona fide* CSC marker for GBM.

CSCs appear to be ideal targets for immunotherapeutics including CAR T cells [8, 13, 14] because, in contrast to more differentiated tumor cells, they are crucial for tumor growth and invasion and for the initiation of local recurrences and distant metastases. However, although we observed CAR T cell-mediated killing of AC133+ patient-derived GBM-SCs *in vitro* and obtained evidence for antitumoral effects of our AC133-CAR T cells in an orthotopic GBM-SC-based glioma mouse model *in vivo*, we did not observe complete cures of the AC133-CAR T cell-treated mice. There are several possible explanations for the lack of sustained and complete tumor eradication in our orthotopic glioma model: (i) The human T cells may not survive long enough in the nude mouse brain due to rejection by residual murine immune cells [36]. (ii) NCH421k GBM-SCs form highly invasive brain tumors [24] and the locally injected CAR T cells may not be able to reach all the invasive GBM-SCs; however, invasive tumor cells might be more efficiently reached if the CAR T cells are transferred into postoperative resection cavities. (iii) Solid tumors are generally more challenging targets for CAR T cells than hematological malignancies, apparently mainly because of the very immunosuppressive microenvironment in solid tumors [8, 11].

There are several different facets of tumor-induced immunosuppression, encompassing immunosuppressive regulatory leukocytes (such as regulatory T cells, myeloid-derived suppressor cells, and M2 macrophages), inhibited dendritic cell maturation, an array of inhibitory cytokines, and immunosuppressive metabolic enzymes, as well as the activation of so-called negative regulatory immune checkpoints [37]. The negative regulatory immune-checkpoint receptors such as CTLA-4 and PD-1 are expressed on T cells upon antigen-specific activation and are further upregulated upon persistent T cell stimulation. Engagement of these receptors by their respective ligands expressed on tumor cells or various tumor stroma cells leads to the gradual loss of T cell effector functions and finally to their physical deletion, a process that is also designated as T cell exhaustion [28]. *In vitro*, we observed only modest upregulation of the major T cell exhaustion marker PD-1 on AC133-CAR T cells upon incubation with AC133+ tumor cells. This does, however, not exclude upregulation of PD-1 on AC133-CAR T cells in the orthotopic glioma xenografts *in vivo*. And, as has also already been shown for CAR T cells, blockade of PD-1 by antagonistic antibodies may significantly improve the effectivity of CAR T cells *in vivo* [38].

Another consequence of chronic antigen-specific stimulation of T cells is terminal T cell differentiation. Terminally differentiated T cells lose their multipotency (they can no longer produce memory or minimally differentiated effector cells), telomeres/telomerase activity and the associated proliferative capacity, and display an increased propensity to apoptosis. However, they usually have a high killing activity and are able to secrete large amounts of effector cytokines [19, 28, 39]. In humans, terminally differentiated T cells are marked by the expression of CD57; its expression increases with age and is associated with chronic infections such as cytomegalovirus or HIV infection and with advanced malignancies. Truly terminally differentiated T cells usually lose expression of the positive co-stimulatory receptors CD28 and CD27 [19, 28]. Recently, however, it has been described that CD57 can also be expressed on incompletely differentiated CD8+ TILs, which may represent a pre-stage to terminal T cell differentiation [33]. These CD57+ CD8+ TILs still express CD28 and CD27, but quickly lose expression of these markers upon further antigenic stimulation. We found that CD57 can be upregulated very quickly on activated T cells upon direct contact with CD57+ target cells, without immediate loss of CD27 or CD28 or their loss upon further antigenic stimulation for several days. Thus, it appears that increased CD57 expression on T cells can also simply reflect an encounter with CD57+ target cells. This may be particularly relevant for T cells specific for tumor entities derived from tissues known to be CD57 positive, such as those derived from the neural crest, particularly the glial and melanocytic lineages [33, 40-42].

One important limitation in CAR T cell therapy is the paucity of truly tumor-specific cell surface target molecules [8, 11]. AC133 is not only expressed on many different types of CSCs but also on normal tissue stem cells such as a subpopulation of CD34+ hematopoietic stem cells [43]. However, postnatally, AC133 seems not to be expressed on normal stem cells in the brain, which may be a good prerequisite for local adoptive transfer in the brain [44]. Nevertheless, adoptive transfer therapy with AC133-CAR T cells may only be possible either by modifications that enhance the target cell specificity (e.g., by using so-called combinatorial or trans-signaling CAR strategies, for which AC133/CD133-specific scFvs have been proposed to be useful [45, 46], or by using an additional CAR with an inhibitory receptor with specificity for normal tissue antigens [47]) or by modifications that allow the rapid elimination of the CAR T cells, if required, such as suicide genes [48].

In summary, we show here that AC133-specific CAR T cells are effective against GBM-SCs both *in vitro* and *in vivo*. Our data suggest that targeting AC133/CD133+ GBM-SCs with CAR T cells has therapeutic potential; however, the potential normal tissue toxicity of such CAR T cells and measures to ameliorate it require further evaluation. In addition, we found that cell-to-cell contacting with patient-derived CD57+ glioma cells induced rapid upregulation of CD57 on activated CD8+ T cells, independent of antigen recognition and without concomitant loss of CD28 or CD27. This finding suggests that CD57 expression does not always reflect terminal or near-terminal T cell differentiation.

## MATERIAL & METHODS

### Cell lines

The glioma cell line U251 was purchased from ATCC, and based on this cell line, a CD133-overexpressing line (CD133-OE U251) was generated by transduction with a lentivirus encoding the human CD133 protein [24]. The NCH421k-, GBM4-, and GBM22-SCs are short-term cultured tumor SC lines derived from GBM tissue and were established and cultured as described previously [24, 34]. The NCH421k-SCs were transduced with a firefly luciferase-encoding lentiviral vector constructed in the laboratory of G.N. [24].

### γ-Irradiation

Irradiations were performed using a Gammacell 40 ^137^Cs laboratory irradiator.

### Plasmid construction

The complete nucleotide sequence of the AC133-specific CAR was designed according to a published ERBB2-specific CAR sequence [49]. We exchanged the single-chain antibody (scFv) in the construct with the AC133-specific scFv, which was derived from the anti-AC133.1 mAb (ATCC HB-12346). The AC133-specific CAR cDNA was synthesized by GenScript. The T2A plus Puro^R^ sequence was cut from pCDH-EF1-MCS-T2A-Puro (System Biosciences) and inserted together with the AC133-CAR into the *piggyBac* transposon vector PB-EF1-MCS-IRES-Neo (System Biosciences) to replace the MCS-IRES-neo part according to standard procedures.

### Stable transfection of T cells with the AC133-CAR

Fresh buffy coats from healthy donors were collected from the Blood Donation Center of the University Hospital Freiburg, and PBMCs were isolated by Pancoll (Pan Biotech) density gradient centrifugation. We followed recently published protocols [12, 50] for stable transfection of PBMCs with *piggyBac* vectors, except for the methods for stimulation and expansion of T cells. Briefly, 10 μg AC133-CAR *piggyBac* transposon vector and 5 μg Super *piggyBac* transposase (System Biosciences) plasmid were used for one nucleofection reaction. To stimulate T cells, on days 1 and 14, allogeneic PBMCs from five donors were irradiated with 40 Gy and added to the T cell culture at a ratio of 10:1 and the cell culture was supplemented with 50 ng/mL of the anti-CD3 mAb OKT3 and 300 IU/mL IL-2. At 2 days after stimulation, the T cells were washed and cultured in T cell medium (RPMI 1640 plus 10% FBS, penicillin-streptomycin, and GlutaMAX) supplemented with 300 IU/mL IL-2 for expansion. The medium was refreshed every 2–3 days. AC133-CAR-expressing T cells were selected with 1 μg/mL puromycin starting 5 days after transfection. Transfected T cells were analyzed on day 24 (10 days after the second stimulation). Nontransfected T cells were generated as described above, but without nucleofection.

### Cytokine production of CAR T cells

In 96-well plates, 2 × 10^5^ AC133-CAR-expressing T cells or activated (nontransfected) control T cells were co-cultured with 1 × 10^5^ U251 WT cells, CD133-OE U251 cells, or NCH421k GBM-SCs, in 200 μL T cell medium per well. After 24 h, the supernatant was collected and the production of IFN-γ was analyzed using Human IFN gamma ELISA Ready-SET-Go (eBioscience) following the manufacturer's instruction.

### Flow cytometric analysis

Cells were washed with PBS containing 0.5% BSA and 2 mM EDTA. Before staining, the cells were incubated with human FcR blocking reagent (Miltenyi Biotec) for 10 min at 4 °C. Then, the cells were stained with specific antibodies or corresponding isotype control antibodies. Thereafter, the cells were washed and analyzed by flow cytometry on a FACSVerse instrument (BD Bioscience).

To determine AC133-CAR-positive cells after transduction, cells were collected 10 days after the second stimulation, washed and stained with mouse anti-c-Myc antibody (clone 9B11; New England Biolabs) followed by a secondary rabbit anti-mouse IgG F(ab')_2_ antibody (Jackson Immunotech).

AC133-CAR T cell phenotype characterization was performed using the following antibodies: anti-CD8, anti-CD27, anti-CD28, and anti-perforin (Miltenyi Biotec); anti-CD45RO and anti-CD62L (eBioscience); anti-PD1 (BioLegend). Determination of AC133 expression on glioma tumor cells was performed with mouse anti-human CD133/1 (Miltenyi Biotec). Anti-CD57 mAbs (clone HNK-1 from BioLegend or clone TB03 from Miltenyi Biotec) were used to detect the expression of CD57 on T cells and glioma cells. In most experiments, T cells were co-cultured with different glioma cells that had been irradiated with 70 Gy. For short-term co-cultures (less than 24 h), the target cells were not irradiated. Co-cultures were incubated at a T cell/tumor cell ratio of 2:1. The T cell population was analyzed at various time points after co-incubation. T cell identification was based on SSC/FSC discrimination and subsequent gating on CD8+ events.

### T cell proliferation assay

T cells were labeled with 0.5 μM CFSE (eBioscience) following the manufacturer's protocol. Target cells were irradiated with 70 Gy, and 1 × 10^6^ CFSE-labeled T cells were co-cultured in a 24-well plate with 5 × 10^5^ irradiated target cells in 1 mL T cell medium supplemented with 300 IU/mL IL-2 per well. After 4 days, T cell proliferation was analyzed by flow cytometry.

### Cytotoxicity assay

U251 WT or CD133-OE U251 cells or NCH421k GBM-SCs were labeled with PKH26 (Sigma) following the manufacturer's instructions. In a 96-well plate, 2 × 10^4^ labeled target cells were seeded. AC133-specific or control T cells were added at different effector/target cell ratios. After 24 h, the cells were collected and stained with 7-AAD (BD Biosciences) and analyzed by flow cytometry. The percentage of lysis was calculated according to the formula: (number of PKH26+ 7-AAD+ cells/number of all PKH26+ cells) × 100%.

### Animal experiments

All animal experiments were performed in accordance with the German Animal License Regulations and were approved by the animal care committee of the Regierungspraesidium Freiburg (registration numbers G-10/64, G-13/12, and G-13/101).

### Orthotopic mouse model of GBM

For intracranial tumor implantation, 6–8-week-old male NMRI nude mice (Harlan) were used. Luciferase-expressing NCH421k GBM-SCs were injected as described before [24]. The BLI signals from tumors were measured using an IVIS spectrum imaging system (Perkin Elmer) every other day. Mice were anesthetized and received 150 mg/kg d-luciferin intraperitoneally. At 1 day before treatment (day 6), the BLI signals usually were between 10^5^ and 10^6^ photons/s. On days 7, 11, and 15 after tumor cell injection, 2 × 10^6^ AC133-specific CAR T cells or nontransfected T cells were injected via the original injection hole, as described above. BLI signals were continually monitored thereafter. Animals were regularly checked for health conditions and sacrificed if they had lost 20% of their body weight or showed signs of neurological deficits.

### *In vitro* differentiation of GBM-SCs

NCH421k GBM-SCs were cultured in DMEM medium containing 10% FBS, supplemented with 1 μM all-*trans*-retinoic acid (Sigma) for 14 days.

### Western blot analysis

Whole-cell lysates of tumor cells were processed for SDS-PAGE and blotting according to standard methods, as described previously [24]. Western blots were probed with antibodies against AC133 (Miltenyi Biotec), SOX-2 (R&D Systems), GFAP (Cell Signaling), actin (Santa Cruz Biotechnology), and HRP-conjugated secondary antibodies (Dianova).

### Statistical analysis

All the calculations were performed using GraphPad Prism software version 6.0 (GraphPad Software Inc.). Animal survival was plotted according to the Kaplan-Meier method and analyzed by log-rank test.
